# Caffeine Supplementation Improves Anaerobic Performance and Neuromuscular Efficiency and Fatigue in Olympic-Level Boxers

**DOI:** 10.3390/nu11092120

**Published:** 2019-09-05

**Authors:** Alejandro F. San Juan, Álvaro López-Samanes, Pablo Jodra, Pedro L. Valenzuela, Javier Rueda, Pablo Veiga-Herreros, Alberto Pérez-López, Raúl Domínguez

**Affiliations:** 1 Laboratorio de Biomecánica Deportiva, Departamento de Salud y Rendimiento Humano, Facultad de Ciencias de la Actividad Física y del Deporte, Universidad Politécnica de Madrid, 28040 Madrid, Spain; 2 School of Physiotherapy, Faculty of Health Sciences, Francisco de Vitoria University, 28223 Madrid, Spain; 3 Faculty of Health Sciences, Alfonso X El Sabio University, 28691 Villanueva de la Cañada (Madrid), Spain; 4 Department of Systems Biology, University of Alcalá, 28805 Madrid, Spain; 5 Departamento de Nutrición Humana y Dietética, Facultad de Ciencias de la Salud, Universidad Alfonso X El Sabio, 28691 Villanueva de la Cañada (Madrid), Spain; 6 Department of Biomedical Sciences, Faculty of Medicine and Health Sciences, University of Alcalá, 28805 Madrid, Spain; 7 Facultad de Ciencias de la Salud, Universidad Isabel I, 09003 Burgos, Spain

**Keywords:** anaerobic, caffeine, CMJ, ergogenic aids, exercise, nutrition, sport supplement, Wingate, electromyography, efficiency

## Abstract

Background: this study examined the effects of caffeine supplementation on anaerobic performance, neuromuscular efficiency and upper and lower extremities fatigue in Olympic-level boxers. Methods: Eight male athletes, members of the Spanish National Olympic Team, were enrolled in the study. In a randomized double-blind, placebo-controlled, counterbalanced, crossover design, the athletes completed 2 test sessions after the intake of caffeine (6 mg·kg^−1^) or placebo. Sessions involved initial measures of lactate, handgrip and countermovement jump (CMJ) performance, followed by a 30-seconds Wingate test, and then final measures of the previous variables. During the sessions, electromiography (EMG) data were recorded on the gluteus maximus, biceps femoris, vastus lateralis, gastrocnemius lateral head and tibialis anterior. Results: caffeine enhanced peak power (6.27%, *p* < 0.01; Effect Size (ES) = 1.26), mean power (5.21%; *p* < 0.01; ES = 1.29) and reduced the time needed to reach peak power (−9.91%, *p* < 0.01; ES = 0.58) in the Wingate test, improved jump height in the CMJ (+2.4 cm, *p* < 0.01), and improved neuromuscular efficiency at peak power in the vastus lateralis (ES = 1.01) and gluteus maximus (ES = 0.89), and mean power in the vastus lateralis (ES = 0.95) and tibialis anterior (ES = 0.83). Conclusions: in these Olympic-level boxers, caffeine supplementation improved anaerobic performance without affecting EMG activity and fatigue levels in the lower limbs. Further benefits observed were enhanced neuromuscular efficiency in some muscles and improved reaction speed.

## 1. Introduction

Caffeine is one of the five nutritional supplements considered ergogenic aids (EA) with good to strong evidence of benefits in specific sports scenarios [1,2], along with other EA such as beetroot juice, sodium bicarbonate, β-alanine, and creatine. All are included in the classification system for nutritional supplements of the Australian Institute of Sports (AIS) based on the demonstrated level of scientific evidence (Level A) [3]. Briefly, the ergogenic effect of caffeine on sports performance can be attributed mainly to: 1) central nervous system stimulation (i.e., blockade of adenosine receptors and release of neurotransmitters such as dopamine, catecholamine and acetylcholine, improving cognitive processes: surveillance, learning, attention and reaction time) [4,5,6], and 2) enhancement of muscle contraction (i.e., improved calcium output from the sarcoplasmic reticulum to the sarcoplasm after the muscle action potential, and increased recruitment of motor units) [7,8,9]. 

There is clear consensus in the literature regarding the effects of caffeine consumption on aerobic performance [10,11]. While fewer studies have focused on sports modalities inducing a predominantly anaerobic metabolism than one mostly dependent on oxidative processes, it is now emerging that caffeine may also have an ergogenic effect on anaerobic efforts [12,13].

The characteristics of combat sports are similar to those of other sports modalities including intermittent dynamics (i.e., high-intensity efforts interspersed with periods of low-intensity activity) [14]. Therefore, at the energy level, combat sports require an important contribution of both aerobic (i.e., oxidative phosphorylation) [15] and anaerobic metabolism (i.e., glycolysis and phosphagen system) during high-intensity actions [16]. Also, combat sports athletes require high levels of isometric handgrip strength [17,18] and muscular endurance in the upper and lower extremities [19]. Competition analysis has revealed that maintenance of power performance during combats is crucial for high-performance in these athletes [20]. 

As combats sports are characterized by high-intensity power actions and both aerobic and anaerobic energy metabolism systems are required, caffeine could be an EA in these sport modalities. However, the effect of this supplement on combat sport performance or fatigue levels has not yet been addressed in the literature. The present study was therefore designed to examine the effects of caffeine supplementation on anaerobic performance, neuromuscular efficiency and neuromuscular fatigue levels in the upper and lower limbs in Olympic-level boxers. We hypothesized that caffeine supplementation would improve anaerobic performance in a 30-seconds all-out Wingate test, improving muscular efficiency without inducing greater mechanical or neuromuscular fatigue.

## 2. Materials and Methods

### 2.1. Participants Selection: Inclusion and Exclusion Criteria

Eight young, healthy male athletes, members of the Spanish National Olympic Team for the Tokyo 2020 Olympic Games (age: 22.0 ± 1.778 years, height: 1.69 ± 0.09 m, body-mass: 65.63 ± 10.79 kg, Body Mass Index (BMI): 22.69 ± 1.31, load Wingate test: 4.91 ± 0.82 kp), were enrolled in the study. 

Exclusion criteria were: (1) age younger than 18 years, (2) having consumed any substance that could affect hormone levels or sport performance in the previous 3 months such as nutrition complements or steroids, (3) having consumed narcotic and/or psychotropic agents, drugs or stimulants during the test or supplementation period, and (4) being diagnosed with any cardiovascular, metabolic, neurologic, pulmonary or orthopedic disorder that could limit performance in the different tests.

At the study outset, participants were informed of the study protocol, schedule and nature of the exercises and tests to be performed before signing an informed consent form. The study protocol adhered to the tenets of the Declaration of Helsinki and was approved by the Ethics Committee of the Alfonso X El Sabio University.

### 2.2. Experimental Design

**Figure 1:**
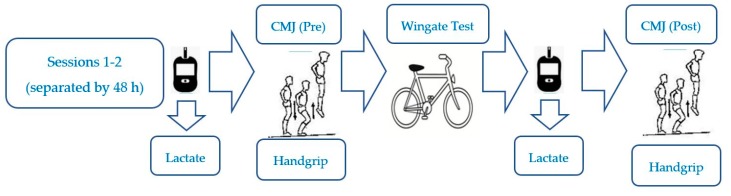
Experimental design. CMJ = countermovement jump test.

A randomized double-blind, placebo-controlled, counterbalanced, crossover design was used in this study. The participants completed 2 identical assessment sessions (see Figure 1) in the laboratory at the same time slot (±0.5 hours) to avoid the detrimental effects of performance associated with circadian rhythm [21]. The test sessions started with initial measures of lactate, handgrip and countermovement jump (CMJ) performance, followed by a 30-seconds Wingate test, and then final measures of the previously collected variables (see Figure 1).

The two sessions were separated by 48 hours. Over a period of 48 hours before the start of the first session until the end of the study, subjects were instructed to follow a series of nutrition requirements and refrain from any type of physical exercise.

### 2.3. Supplementation and Diet Control

The authors packaged and prepared the capsules containing caffeine or placebo (sucrose). The capsules used were no.1 opaque red (Guinama S.L.U, 0044634, La Pobla de Valbona, Spain). For the encapsulation process, we followed the normalized working procedures described for this purpose [22]. The filling equipment used was a manual semiautomatic Carsunorm 2000 system (Miranda de Ebro, Spain).

The subjects arrived at the laboratory 75 minutes before the start of the session, when they were given a capsule containing either a caffeine supplement (6 mg·kg^−1^) or sucrose (6 mg·kg^−1^, placebo). Caffeine dosage selection (6 mg·kg^−1^) was made to promote the higher ergogenic effects producing the minimum side-effects possible [1]. The protocol timing was designed considering that caffeine reaches peak concentrations in blood after 1 hour of intake [23], and the degradation quality control tests its half-life (13.4 minutes) according to previous description [22].

In addition, participants received dietary guidelines to ensure that they all followed a diet with the same content of macronutrients (i.e., 60% of energy intake in the form of carbohydrates, 30% lipids and 10% proteins) in the 48 hours prior to each session. A list of foods rich in caffeine was provided to all participants (e.g., coffee, tea, mate, tea soft drinks, energy drinks, cola drinks, chocolate drinks and chocolate) so that they avoided caffeine intake from 24 hours before the study to the end of the study.

### 2.4. Wingate Test

A 30-seconds all-out Wingate test was performed on a Monark cycloergometer (Ergomedic 828E, Vansbro, Sweden). Before the test, a warm-up protocol was conducted consisting of 5 minutes pedaling at low intensity (i.e., subjects chose the load and cadence), followed by another 5 minutes pedaling at 60 revolutions per minute (rpm) with a load of 2 kiloponds (Kp). In the last 5 seconds of each minute, the subjects performed a maximum intensity sprint. After three minutes, subjects performed three countermovement jumps (CMJs) at increasing intensity with 10 seconds recovery between jumps. Then, 2 CMJs were executed on the force platform. After two minutes of recovery, the Wingate test began. Subjects pedaled as fast as possible for 30 seconds against a constant load (Kp) calculated according to the 7.5% of each participant body mass [24]. The instructions given to them were: i) reach maximum rpm in the shortest time and ii) try to keep the highest number of rpm until the end of the test. During the test, subjects were encouraged by 4 researchers from the beginning until the end. Power output (W) was analyzed during each second and, later, peak power output (W_peak_), time (s) to reach W_peak_ (TW_peak_), mean power output during the 30 seconds sprint (W_mean_) and minimum power output during the last 10 seconds of the test (W_min_) were calculated. In addition to W_mean_ during the entire sprint, mean power output was also calculated every 5 seconds of the sprint (Split_1-5S_, Split_6-10S_, Split_11-15S_, Split_16-20S_, Split_21-25S_, Split_26-30S_).

### 2.5. Electromyographic Assessment

Electromiography (EMG) data were recorded from the following muscles: gluteus maximus (GM), biceps femoris (BF), vastus lateralis (VL), gastrocnemius lateral head (GL), and tibialis anterior (TA) and the mean of the five muscles analyzed (MED). We used a “Trigno Wireless SystemTM Delsys” (Delsys Inc. Massachusetts, MA, USA). Briefly, one active electrode was placed on the bellies of each muscle of the right thigh and leg following the protocol established by the SENIAM Project (Surface ElectroMyoGraphy for the Non-invasive Assessment of Muscles) [25]. These electrodes recorded the surface electrical activity corresponding to the underlying muscle, sampled at a frequency of 1024 Hz. The EMG signal was filtered by a band pass between 20 and 300 Hz, and subsequently the EMG Root Mean Square signal (rms-EMG) was calculated. The rms-EMG variable obtained from each of the 5 muscles was normalized to the maximum value obtained in the corresponding muscle for 1 second. In our study, rms-EMG was used as an estimate of “total myoelectric activity” of the exercising muscle as it has been previously shown that this computation: 1) is an accurate measure of EMG amplitude and 2) is highly correlated with the number of active motor units (fiber recruitment) [26,27].

To facilitate the analysis of results, the 30 seconds of each Wingate test was divided into groups of 5 seconds and we calculated the rms-EMG mean in this time period (e.g., EMG_0–5s_, EMG_6–10s_, EMG_11–15s_). In addition, we calculated the average rms-EMG (EMG_mean_), the rms-EMG corresponding to the time where W_peak_ was reached (EMG_Wpeak_), the time (s) to reach the rms-EMG peak record (TEMG_peak_) and the rms-EMG corresponding to the time when W_min_ was reached (EMG_Wmin_). Data of rms-EMG is expressed as a base index one where the value 1 is equal to 100% (i.e., the value 0.75 is equal to 75 %).

Additionally, to analyze neuromuscular efficiency (NME), we used the ratios between W_peak_ and EMG_Wpeak_ (NME_Wpeak_) and between W_mean_ and EMG_Wmean_ (NME_Wmean_). Neuromuscular efficiency (NME) was used as an index of neuromuscular fatigue [28] and was estimated from the ratio of power to non-normalized RMS (raw EMG data in volts). We adapted the methodology described by Hug and Dorel [28], and we propose a ratio of power output to normalized RMS (EMG data in percent of muscle activation). Our rationale was that to determine NME, it is better to relate power to percent of motor units activated than to raw volts, as described in the literature, and more often used as a measure of fatigue [28].

### 2.6. Blood Lactate

Before the warm-up period and immediately after the Wingate test, 5 μ·l samples of capillary blood from the soft part of the index finger of the left hand were obtained and subjected to blood lactate concentration determination using a Lactate ProTM 2 LT-1710 blood analyzer (Arkray Factory Inc., KDK Corporation, Shiga, Japan).

### 2.7. Neuromuscular Fatigue

Neuromuscular fatigue in the lower limbs was measured in a CMJ [29] performed on a force platform (Quattro Jump model 9290AD; Kistler Instruments, Winterthur, Switzerland). Before the jump was initiated, participants stood on the platform with legs extended and hands on hips. For the jump, the legs were first flexed to 90º (eccentric action) and then explosively extended in a coordinated manner (concentric action) trying to reach maximum height. During the flight stage, the knees were extended. Contact with the ground was made with the toes first. During the test, subjects were instructed to keep their hands on their hips and avoid any sideways displacements during the flight stage. This same protocol was applied for the CMJs performed before and after the Wingate test.

From each CMJ test, jump height, mean (CMJ_Wmean_) and peak power produced (CMJ_Wpeak_) were extracted, as indicators of neuromuscular fatigue [30].

### 2.8. Handgrip Strength

Isometric handgrip strength (IHS) was measured twice for the dominant hand using a calibrated handgrip dynamometer (Takei 5101, Tokyo, Japan) with 30 seconds of passive recovery between trials. Participants sat with 0 of shoulder flexion and elbow flexion, and the forearm and hand in a neutral position and exerted their maximal strength during 5 seconds [31]. The highest value of the dominant hand was recorded and used for statistical analysis as the maximum voluntary handgrip strength. 

### 2.9. Statistical Analysis

Results for all parameters are presented as mean ± standard deviation (SD). Data analyses were carried out using the commercial software “Statistical Package for Social Sciences” SPSS v21.0 software (SPSS Inc., Chicago, IL, USA). The effects of caffeine supplementation on Wingate test performance, lactate, CMJ and strength grip performance were assessed through a two-way ANOVA test for condition (caffeine versus placebo) and time (pre-versus post-Wingate for CMJ handgrip strength measures, and during each 5 seconds period of the Wingate test). Levene’s test revealed the homogeneity of variances of the data and the Shapiro-Wilk’s test confirmed their normal distribution. When a significant main effect was detected, pairwise comparisons were assessed using the Holm-Bonferroni test in order to ensure protection against multiple comparisons. Additionally, W_peak_, TW_peak_, W_mean_, W_min_, EMG_Wpeak_, TEMG_max_, EMG_mean_ and EMG_Wmin_ and efficiency measures (NME_Wpeak_, NME_Wmean_ and NME_Wmin_) were analyzed using the Student’s t-test. Pairwise comparisons significance was assessed by calculating Cohen’s d Effect Size (ES) [32]. Effect sizes (d) above 0.8, between 0.8 and 0.5, between 0.5 and 0.2 and lower than 0.2 were considered as large, moderate, small, and trivial, respectively [33,34].

## 3. Results

### 3.1. Wingate Test

Compared to placebo, caffeine consumption produced a significant and large effect in W_peak_ (10.84 ± 0.49 versus 10.20 ± 0.59; *p* < 0.01; Effect Size (ES) = 1.26) and W_mean_ (8.68 ± 0.34 versus 8.25 ± 0.37; *p* < 0.01; ES = 1.29), a decrease in TW_peak_ (8.00 ± 1.60 versus 8.88 ± 1.64; *p* < 0.01; ES = 0.58), while this improvement after caffeine supplementation in W_min_ it was not significantly different (*p* = 0.123) (see Table 1). Moreover, there was an effect of the time factor (*p* < 0.001), verified in the analysis of power output levels throughout the 6 partial tests, as well as for the supplementation factor (*p* = 0.006). Significant differences were observed in Split_6–10s_ (*p* = 0.026) and Split_11–15s_ (*p* = 0.009), as well as a significant trend Split_16–20s_ (*p* = 0.062) (see Table 2). There was no significant interaction between factors (supplementation-time).

### 3.2. Electromyographic Assessment and Neuromuscular Efficiency

In the analysis of rms-EMG, there were no significant differences (*p* > 0.05) between supplementation in EMG_Wpeak_, EMG_mean_ and EMG_Wmin_ during the Wingate test (see Table 1). Also, we observed a higher TEMG_max_ in the gluteus maximus for the caffeine condition (8.00 ± 6.26 versus 3.63 ± 3.66; p = 0.022; ES = 0.91). 

On the other hand, there was a time factor effect in EMG_VL_, EMG_BF_, EMG_TA_, EMG_GL_ (*p* < 0.05), in the placebo and caffeine conditions at different Wingate time splits (see Table 2). There were no significant differences for supplementation conditions or the interaction between factors (supplementation-time) (*p* > 0.05), except for EMG_TA_ (time·suplementation: *p* = 0.033).

In the analysis of neuromuscular efficiency there were no significant differences between caffeine and placebo conditions, but a large effect was detected for NME_Wpeak_ in the vastus lateralis (ES = 1.01) and gluteus maximus (ES = 0.89), and NME_Wmean_ for vastus lateralis (ES = 0.95) and tibialis anterior (ES = 0.83). There was also a moderate effect near large values (i.e. ≈ 0.8), in NME_MED_ at W_peak_ (ES = 0.77), and at W_mean_ (ES = 0.74) (see Table 3).

### 3.3. Blood Lactate

**Figure 2:**
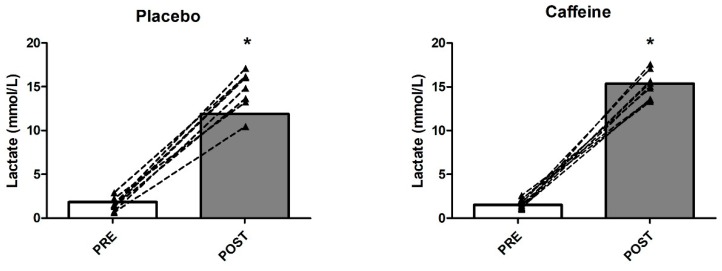
Blood lactate concentrations pre-post Wingate. * *p* < 0.05, significant differences compared to pre-Wingate values (PRE).

Blood lactate concentrations increased from rest (placebo 1.86 ± 0.55 mmol·L^−1^ versus caffeine 1.53 ± 0.56 mmol·L^−1^ ) to exhaustion after the Wingate test (placebo 11.88 ± 1.55 mmol·L^−1^ versus caffeine 15.36 ± 1.57 mmol·L^−1^ ), with significant differences in the placebo (*p* < 0.001) and caffeine conditions (*p* < 0.001), but not between conditions (*p* > 0.05) (see Figure 2). 

### 3.4. Neuromuscular Fatigue (CMJ) and Handgrip Strength

Before the Wingate test, caffeine consumption increased jump height (Placebo versus Caffeine, 43.1 ± 3.7 versus 45.4 ± 4.2 cm; *p* = 0.006), but not CMJ_Wmean_ (Placebo versus Caffeine, 28.8 ± 3.0 versus 29.1 ± 4.9 W; *p* > 0.05) or CMJ_Wpeak_ (Placebo versus Caffeine, 51.3 ± 3.4 versus 51.6 ± 5.7 W; *p* > 0.05). The analysis of the CMJs performed before and after the Wingate test revealed a significant decrease in jump height, CMJ_Wmean_ and CMJ_Wpeak_ after caffeine and placebo ingestion (ANOVA time effect, *p* = 0.001). Although compared to placebo, caffeine promoted a less pronounced decrease in jump height, CMJ_Wmean_ and CMJ_Wpeak_ (−2.5%, −1.3% and −2.0%, respectively) only jump height showed a difference between conditions (ANOVA effect, *p* = 0.020). In the analysis of handgrip strength, there were no differences detected for supplementation, time or time·supplementation (*p* > 0.05).

## 4. Discussion

Our results show that the ingestion of caffeine in Olympic-level boxers significantly improves anaerobic performance and has a positive effect on neuromuscular efficiency. Caffeine was also found to reduce lower limbs fatigue levels after an anaerobic test. To our knowledge, this is the first study that has examined the effects of caffeine in Olympic-level boxers. 

The main findings of the present study were that caffeine supplementation (6 mg·kg^−1^) enhanced W_peak_ (6.27%, *p* < 0.01; ES = 1.26) and W_mean_ (5.21%; *p* < 0.01; ES = 1,29) and reduced TW_peak_ (−9.91%, *p* < 0.01; ES = 0.58) in the Wingate test, improved jump height in the CMJ (+2.4 cm, *p* < 0.01) and showed a large effect on neuromuscular efficiency, improving NME_Wpeak_ in the vastus lateralis (ES = 1.01) and gluteus maximus (ES = 0.89) and NME_Wmean_ for the vastus lateralis (ES = 0.95) and tibialis anterior (ES = 0.83). Thereby, these results are in accordance with the 21 meta-analysis review conducted by Grgic et al. [35], who stated that ingestion of caffeine enhanced a large span of exercise performance variables (e.g., muscle endurance and strength, anaerobic power).

Our results with caffeine ingestion seem to improve the most important physical capacities for elite level boxers [36] (e.g., maximal strength and power output, muscle resistance). We observed significant improvements in peak power (6.27%) and mean power (5.21%) in the Wingate test, and in CMJ jump height (5.1%). These findings are consistent with a meta-analysis that have found similar results for peak and mean power [12] and power production [13]. Also, these results are competitively relevant because improvements around 0.6% are enough to make a difference in elite-level sports [37,38]. 

During the Wingate test, boxers in both conditions, generated the highest power during the second split (6–10 seconds) and then power production decreased progressively until the end of the test. In a Wingate test, W_peak_ is commonly reached during the first 6 seconds of the sprint where free adenosine triphosphate (ATP) and phosphocreatine (PCr) stores are essential energy sources [24,39]. Accordingly, during the 5–10 seconds of the sprint the critical reduction of PCr pools in the muscle promotes adenosine diphosphate (ADP) accumulation which causes the end of the exercise [40]. Given the physiological characteristic of boxing, the delayed time to reach W_peak_ in elite boxers may be explained by an increased capacity to store PCr in their muscles. Further, the caffeine condition showed a higher mean power output in all the splits (differences in splits ranged from +0.27 to +0.56 W·kg^−1^). These data support the conclusion reached in two caffeine meta-analyses [12,13] where the ergogenic effect of this supplement was attributed to the capacity to improve the production of power by skeletal muscle.

Another main result observed in the present study is the higher neuromuscular efficiency (NME) measured by superficial EMG during the Wingate test in the caffeine condition. To our knowledge, this is the first attempt to asses this question in Olympic level boxers. Mean EMG recordings were similar between both conditions (*p* > 0.05). However, as we described before, the caffeine condition showed a significantly improved power released (i.e., peak power (+0.64 W·kg^−1^) and time to achieve peak power (−0.88 seconds), and mean power (+0.43 W·kg^−1^)), so the boxers in the caffeine condition developed higher power with the same muscle activation (i.e., greater NME). Moreover, we observed a moderate effect near to large values (i.e., ES > 0.8) for the five muscles measured together NME_MED_ peak (ES = 0.77) and mean (ES = 0.74), and large effect for neuromuscular efficiency (ES > 0.80) for some muscles (i.e., vastus lateralis, gluteus maximus, tibialis anterior). This improved neuromuscular efficiency may be due to the caffeine-enhanced intra- and inter-muscle coordination [41]. Moreover, the vastus lateralis and the gluteus maximum are two of the main muscles involved in pedaling, mostly in the down-stroke phase [42]. Further, in vitro findings observed the increase in calcium release from the sarcoplasmic reticulum after an action potential that could explain these ergogenic effects [43]. In parallel, the significantly longer time (>4 seconds) to achieve EMG peak in the gluteus maximum (TEMG_peak GM_) in the caffeine condition, also supports this improved neuromuscular efficiency. Then, during the Wingate test, we observed a greater mean power released in the caffeine condition in each 5 seconds split, with global maintained fiber recruitment (even with a tendency towards lower muscle activation), and with a delay to achieve peak muscle activity in one of the most important muscles in cycling, the gluteus maximum. Therefore, it seems that the higher power production and delayed muscle activity of the gluteus maximum caused by caffeine consumption, facilitated an increased time to produce higher power output (>4 seconds) at the beginning of the test. Also, the higher NME of the tibialis anterior, overall an important up-stroke muscle during the Wingate test, may help to produce this higher power output in the caffeine condition. But further, the NME_MED_ of the five muscles contribute to maintain this greater power production during the 30 seconds of the Wingate test with the same muscle recruitment, resulting in better neuromuscular efficiency. This improved duration during high-power actions was observed by Coswig et al. [44] after caffeine supplementation, ten Judo athletes increased the duration of high-intensity actions and decreased the rest duration during simulated boxing matches. However, Greer, Morales, and Coles [45] studied the effects of caffeine ingestion on Wingate performance and surface EMG in eighteen active males. They observed no differences in neuromuscular efficiency (i.e., same power output and EMG amplitude between conditions). This lack of ergogenic effect may be explained because it could be exclusive to athletes with high levels of performance, as there are other studies with poorly trained subjects where there have been no significant differences between caffeine and placebo conditions [46,47,48,49]. More deeply, MacIntosh et al. [50] and Lucia et al. [27] studied this neuromuscular efficiency in cycle ergometry with active healthy subjects and professional elite cyclists, respectively. Both showed that at high power outputs (i.e. ≈ 400 W), higher pedaling cadence produced lower rms-EMG amplitude, and then lower motor unit activation. In the present study, the cycle ergometer was set with the same fixed load for each boxer in both conditions. In the caffeine condition, they produce higher power output with these fixed loads, so caffeine permits a higher pedaling cadence to produce this increased power. Then, this higher cadence may in part explain the better neuromuscular efficiency observed in these elite boxers.

On the other hand, there was a significant EMG fatigue effect in the placebo and caffeine conditions at different Wingate time splits (ANOVA time effect: EMG_VL_, EMG_BF_, EMG_TA_, EMG_GL,_ *p* < 0.05; EMG_GM_ *p* = 0.094). The data revealed in the five muscles mean EMG differences of −13.9% ± 7.0% (range −4% to −24%) from the first Wingate split (0–5 seconds) to the last (26–30 seconds). The rms-EMG used in the present study is an accurate measure of the EMG amplitude and is highly correlated with the number of active motor units (fiber recruitment) [26,27]. Then, fiber recruitment decreased progressively during the Wingate test influenced by higher fatigue levels. The same behavior was described in the Wingate test by Greer, Morales, and Coles [45]. They measured vastus lateralis and gastrocnemius muscles and observed a significantly decreased EMG amplitude during the 30-seconds all-out test, with no differences between caffeine and placebo conditions. In combat sports, Cortez et al. [51] observed the same fatigue effect at the level of the rectus femoris in a dollyo chagi kick (i.e., Taekwondo technique), before and after a strenuous task, and observed that caffeine supplementation reduced this fatigue effect compared to the intake of placebo (≈ −5% Caffeine versus ≈ −20% Placebo). 

Caffeine has shown to be effective at improving reaction speed (i.e., reducing the execution time of the bandal tchagui kick) [52], or reaction time in response to a visual stimulus [53], in combat sports. Although, the reaction speed of the upper extremities has not been measured, the shorter time to reach the achieve peak power during the Wingate test (~10%) seems to support this ergogenic effect of caffeine in boxers. This effect of caffeine intake on reaction speed could be mediated by increased neurotransmitter delivery, enhancing motor neuron transmission [54,55,56], and by increased activity of the sodium-potassium pump, improving the sarcoplasmic availability of calcium [55]. 

In the present study, caffeine consumption enhanced neuromuscular performance and diminished neuromuscular fatigue, measured with the countermovement jump test, by significantly increasing vertical jump height (+2.3 cm) and attenuating the decrease in vertical jump height after the Wingate test (−2.5%) respectively, and compared to placebo. Fatigue is a very important variable in combat sports such as Olympic boxing, as the competitions include multiple fights on consecutive days, and then the maintenance of power levels between fights is considered a valuable performance variable [20]. Our results agree with those of Cortez et al. [51], who observed higher neuromuscular performance and lower levels of fatigue in a dollyo chagi kick (i.e., Taeckwondo technique) in taekwondo athletes supplemented with caffeine, before and after a strenuous task compared to placebo intake. 

Our data showed that an anaerobic effort such as a Wingate Test results in a significant increase in blood lactate concentrations in both conditions (time factor for both placebo and caffeine), but not between conditions (*p* > 0.05). These findings are in agreement with other data published in well-trained men [57], Judo athletes [58], and male wrestlers [20]. However, although we did not find significant differences between conditions, we detected higher lactate concentrations for caffeine supplementation versus placebo. This large amount of lactate production in the caffeine condition may be explained by the observed better Wingate performance, that may reflect a higher glycolysis utilization [12]. Other authors [59,60] observed significantly augmented blood lactate concentrations in taekwondo and Jiu-Jitsu athletes following caffeine supplementation. As before, this effect could be explained by the higher energy expenditures related to increased glycolysis utilization with a greater recruitment of type II motor units [61] (i.e., highly dependent on glycolytic metabolism [62,63]), and by a reduced effect of adenosine on phosphofructokinase inhibition [43].

In comparison to the placebo condition, no differences were noted in the isometric handgrip strength (IHS) with caffeine ingestion (−1.34% versus −0.54%). Our results are similar to previous data with highly resistant training males [64] that reported no differences with caffeine ingestion versus placebo conditions in IHS (1.88%) after a neuromuscular test battery. However, other studies have found improvements in handgrip force after caffeine supplementation [9,60,65,66]. The lack of significant effect of caffeine consumption on isometric strength and the discrepancy observed in the literature may indicate that this ergogenic aid is more effective on dynamic (eccentric and concentric) compared to static (isometric) muscular performance. Moreover, it should take into account that caffeine ingestion stimulates a higher increase in lower body compared to upper body strength performance [67]. Handgrip is not a specific action for boxing athletes and may not be the most appropriate test for them. In fact, we can speculate that another explanation of this result may reside on the differences in muscle recruitment and contraction between the handgrip strength test and how boxers train their hands. While the handgrip strength test required maximal strength of the flexor muscle of the hand, in boxing, other muscles of the forearm are implicated and maximal contraction may not be required. Another explanation may be that the great endurance-strength of these Olympic-level boxers could overcome the fatigue effect of one Wingate test (i.e., focused overall on lower limbs performance). In future studies it should be recommended to determine the ergogenic effects of caffeine on both upper and lower limbs, by subjecting the boxers to several bouts of a specific test that includes the four extremities. In this sense, Negaresh et al. [68], observed during a simulated wrestling tournament that an individualized caffeine supplementation protocol should be implemented when physical performance is expected to be reduced (i.e., usually during the latter combat rounds). 

### Limitations

Due to the high quality of the sample, its number is limited and could have masked some of the known ergogenic effects of caffeine. Moreover, all the enrolled subjects were male. Lastly, blood samples extraction would help to monitor caffeine presence in plasma in both trials (caffeine and placebo), a procedure that cannot be performed in the present cohort of elite boxers. Future studies using a bigger sample with mixed-gender or female population and blood samples are warranted.

## 5. Conclusions

The present study has demonstrated that caffeine supplementation (6 mg·kg^−1^) improves anaerobic performance (i.e., Wingate and CMJ) with a similar electromyographic activity and fatigue levels of lower limbs (i.e., Wingate and CMJ) and enhanced neuromuscular efficiency in some muscles (i.e., vastus lateralis, gluteus maximus and tibialis anterior) in Olympic-level boxers. Further, caffeine consumption enhances reaction speed (i.e., a higher peak power with a lower time to achieve peak power).

Future research should focus on the ergogenic effects of caffeine after repeated bouts of a specific simulated boxing combat test on both the upper and lower extremities and should also address cognitive fatigue.

## Figures and Tables

**Table 1:** Data for power output and root mean square-EMG (rms-EMG) recorded during the Wingate test.

Variable	Experimental Condition	W_peak_-EMG_Wpeak_			TW_peak_-TEMG_peak_			W_mean_-EMG_mean_			W_min_-EMG_Wmin_		
		M ± SD	*p*-Value	ES	M ± SD	*p*-Value	ES	M ± SD	*p*-Value	ES	M ± SD	*p*-Value	ES
**W_output_**	Placebo	10.20 ± 0.59	<0.01 *	1.26	8.88 ± 1.64	0.01 *	0.58	8.25 ± 0.37	0.01 *	1.29	6.19 ± 0.56	0.123	0.75
	Caffeine	10.84 ± 0.49			8.00 ± 1.60			8.68 ± 0.34			6.49 ± 0.22		
EMG_VL_	Placebo	0.78 ± 0.09	0.268	0.71	12.25 ± 9.27	0.270	0.68	0.74 ± 0.11	0.247	0.62	0.41 ± 0.15	0.332	0.47
	Caffeine	0.69 ± 0.17			7.38 ± 5.58			0.66 ± 0.16			0.33 ± 0.21		
EMG_BF_	Placebo	0.67 ± 0.19	0.435	0.29	8.63 ± 3.70	0.292	0.36	0.55 ± 0.14	0.254	0.37	0.26 ± 0.11	0.430	0.37
	Caffeine	0.72 ± 0.18			12.13 ± 8.43			0.60 ± 0.15			0.31 ± 0.17		
EMG_GM_	Placebo	0.68 ± 0.16	0.311	0.73	3.63 ± 3.66	0.022 *	0.91	0.64 ± 0.08	0.728	0.25	0.36 ± 0.31	0.387	0.22
	Caffeine	0.56 ± 0.19			8.00 ± 6.26			0.62 ± 0.09			0.31 ± 0.15		
EMG_TA_	Placebo	0.73 ± 0.21	0.984	0.00	7.75 ± 3.45	0.722	0.16	0.63 ± 0.10	0.298	0.59	0.23 ± 0.12	0.423	0.26
	Caffeine	0.73 ± 0.20			7.13 ± 4.55			0.55 ± 0.18			0.20 ± 0.13		
EMG_GL_	Placebo	0.74 ± 0.15	0.824	0.16	8.00 ± 5.37	0.936	0.05	0.67 ± 0.12	0.935	0.09	0.40 ± 0.11	0.980	0.00
	Caffeine	0.76 ± 0.12			7.75 ± 5.03			0.66 ± 0.13			0.40 ± 0.16		
EMG_MED_	Placebo	0.72 ± 0.07	0.607	0.60				0.65 ± 0.05	0.261	0.44	0.33 ± 0.07	0.343	0.22
	Caffeine	0.69 ± 0.03						0.62 ± 0.09			0.31 ± 0.12		

W_peak_: Peak power (w/kg); EMG_Wpeak_: rms-EMG at W_peak_; TW_peak_: time (s) to achieve the maximal power; TEMG_peak_: time (s) to achieve the maximal rms-EMG record; W_mean_: Average power (w/kg); EMG_mean_: Average rms-EMG; W_min_: Minimum power (w/kg); EMG_Wmin_: rms-EMG at W_min_; EMG_VL_: rms-EMG recorded on the vastus lateralis; EMG_BF_: rms-EMG recorded on the biceps femoris; EMG_GM_: rms-EMG recorded on the gluteus maximus; EMG_GL_: rms-EMG recorded on the gastrocnemius lateral head; EMG_TA_: rms-EMG recorded on the tibialis anterior; EMG_MED_: Mean rms-EMG recorded on the five muscles analyzed; Data of power output expressed as Watts·kg^−1^, and rms-EMG data as a base index one. * Significant difference between Placebo and Caffeine condition at *p* < 0.05.

**Table 2:** Mean and standard deviations (SD) of power output and rms-EMG data during 6 splits in the Wingate Test.

Variable		Split_1–5s_	Split_6–10s_	Split_11–15s_	Split_16–20s_	Split_21–25s_	Split_26–30s_	*p*-Value Time	*p*-Value Supplementation	*p*-Value Time Supplementation
W_output_	Placebo	6.61 ± 0.89 ^#A^	9.98 ± 0.59 ^#D^ *	9.63 ± 0.65 ^#H^ *	8.80 ± 0.64 ^#L^	7.78 ± 0.36 ^#O^	6.68 ± 0.38	<0.001 ^#^	0.006 *	0.696
	Caffeine	7.05 ± 1.11 ^#A^	10.54 ± 0.56 ^#D^	10.19 ± 0.58 ^#H^	9.18 ± 0.70^#L^	8.05 ± 0.56 ^#O^	7.04 ± 0.34			
EMG_VL_	Placebo	0.72 ± 0.10	0.76 ± 0.10	0.79 ± 0.10	0.79 ± 0.14 ^#M^	0.73 ± 0.16	0.67 ± 0.17	0.018 ^#^	0.247	0.985
	Caffeine	0.62 ± 0.19	0.69 ± 0.15	0.72 ± 0.19	0.68 ± 0.16	0.64 ± 0.20	0.58 ± 0.20			
EMG_BF_	Placebo	0.53 ± 0.14 ^#B^	0.75 ± 0.10 ^#E^	0.65 ± 0.16 ^#I^	0.54 ± 0.22 ^#N^	0.43 ± 0.19	0.36 ± 0.16 ^#P^	0.002 ^#^	0.250	0.089
	Caffeine	0.60 ± 0.14	0.71 ± 0.10	0.71 ± 0.16 ^#I^	0.64 ± 0.20 ^#N^	0.52 ± 0.20	0.46 ± 0.19			
EMG_GM_	Placebo	0.73 ± 0.13	0.63 ± 0.12	0.65 ± 0.08	0.65 ± 0.10	0.59 ± 0.05	0.57 ± 0.10	0.094	0.734	0.286
	Caffeine	0.63 ± 0.16	0.59 ± 0.19	0.61 ± 0.15	0.66 ± 0.11	0.66 ± 0.12	0.56 ± 0.11			
EMG_TA_	Placebo	0.61 ± 0.16 ^#B^	0.75 ± 0.16	0.76 ± 0.11 ^#J^	0.65 ± 0.14 ^#M^	0.56 ± 0.11	0.47 ± 0.08 ^#Q^	<0.001^#^	0.298	0.033^₸^
	Caffeine	0.57 ± 0.17	0.70 ± 0.16 ^#F^	0.59 ± 0.22 ^#K^	0.51 ± 0.20	0.45 ± 0.22	0.43 ± 0.24			
EMG_GL_	Placebo	0.77 ± 0.12 ^#C^	0.76 ± 0.09 ^#G^	0.68 ± 0.14	0.65 ± 0.14	0.61 ± 0.16	0.53 ± 0.16	<0.001^#^	0.948	0.592
	Caffeine	0.75 ± 0.11^#C^	0.73 ± 0.08	0.68 ± 0.18	0.70 ± 0.19 ^#M^	0.61 ± 0.19	0.51 ± 0.17			

**EMG_VL_:** rms-EMG recorded on the vastus lateralis; **EMG_BF_:** rms-EMG recorded on the biceps femoris; **EMG_GM_:** rms-EMG recorded on the gluteus maximus; **EMG_GL_:** rms-EMG recorded on the gastrocnemius lateral head; **EMG_TA_:** rms-EMG recorded on the tibialis anterior; Data of power output expressed as Watts·kg^−1^, and rms-EMG data as a base index one. **#**: Significant differences in factor time at *p* < 0.05. *: Significant difference between Placebo and Caffeine condition at *p* < 0.05. **₸**: Significant difference in interaction Time-Supplementation at p < 0.05.Significance differences between splits: ^#**A**^**:** Split_6-10s_, Split_11–15s_ and Split_16–20s_ versus Split_1–5s._^#**B**^**:** Split_6–10s_ versus Split_1–5s._ **^#C^:** Split_26–30s_ versus Split_1–5s._ **^#D^:** Split_0–5s_, Split_16–20s_, Split_21–25s_ and Split_25–30s_ versus Split_6–10s._ **^#E^:** Split_21–25s_, Split_26–30s_ versus Split_6–10s._ **^#F^:** Split_16–20s_, Split_21–25s_, Split_26–30s_ versus Split_6–10s._ **^#G^:** Split_26–30s_ versus Split_6–10s._ **^#H^:** Split_1–5s_, Split_16–20s_, Split_21–25s_ and Split_25–30s_ versus Split_11–15s._ **^#I^:** Split_26–30s_ versus Split_11–15s_^#**J**^**:** Split_21–25s_, Split_26–30s_ versus Split_11–15s_ **^#K^:** Split_21–25s_ versus Split_11–15s._ **^#L^:** Split_1–5s_, Split_6–10s_, Split_16–20s_, Split_21–25s_ and Split_25–30s_ versus Split_16–20s._ **^#M^:** Split_26–30s_ versus Split_16–20s._ **^#N^:** Split_21–25s_, Split_26–30s_ versus Split_16–20s._ **^#O^:** Split_6–10s_, Split_16–20s_, Split_21–25s_ and Split_25–30s_ versus Split_21–25s._^#**P**^**:** Split_6–10s_, Split_11–15s_ and Split_16–20s_ versus Split_26–30s._ **^#Q^:** Split_6–10s_, Split_11–15s_, Split_16–20s_, Split_21–25s_ versus Split_26–30s._

**Table 3:** Data of neuromuscular efficiency for the different muscles analyzed during the Wingate test.

Variable	Experimental Condition	NME_Wpeak_			NME_Wmean_		
		M ± SD	*p*-Value	ES	M ± SD	*p*-Value	ES
**NME_VL_**	Placebo	13.29 ± 1.63	0.115	1.01	11.34 ± 1.98	0.105	0.95
	Caffeine	16.71 ± 4.87			13.99 ± 3.71		
NME_BF_	Placebo	16.75 ± 5.87	0.785	0.12	16.19 ± 5.00	0.678	0.17
	Caffeine	16.11 ± 5.28			15.39 ± 5.30		
NME_GM_	Placebo	15.74 ± 4.01	0.187	0.89	13.14 ± 1.75	0.261	0.61
	Caffeine	22.18 ± 10.19			14.27 ± 2.19		
NME_TA_	Placebo	15.72 ± 7.59	0.957	0.04	13.43 ± 3.06	0.181	0.83
	Caffeine	15.93 ± 4.66			18.11 ± 7.94		
NME_GL_	Placebo	14.47 ± 3.77	0.947	0.04	12.76 ± 2.88	0.556	0.31
	Caffeine	14.58 ± 2.20			13.82 ± 4.35		
NME_MED_	Placebo	14.35 ± 1.99	0.184	0.77	12.87 ± 1.41	0.054	0.74
	Caffeine	15.92 ± 2.34			14.36 ± 2.71		

NME_Wpeak_: ratio between W_peak_ and EMG_Wpeak_; NME_Wmean_: ratio between W_mean_ and EMG_Wmean_; NME_VL_: neuromuscular efficiency measured on the vastus lateralis; NME_BF_: neuromuscular efficiency measured on the biceps femoris; NME_GM_: neuromuscular efficiency measured on the gluteus maximus; NME_GL_: neuromuscular efficiency measured on the gastrocnemius lateral head; NME_TA_: neuromuscular efficiency measured on the tibialis anterior; NME_MED_: neuromuscular efficiency measured as the mean values of the five muscles analyzed; * Significant difference between Placebo and Caffeine condition at *p* < 0.05.
